# Children’s Physical Activity Levels Following Participation in a Classroom-Based Physical Activity Curriculum

**DOI:** 10.3390/children6060076

**Published:** 2019-06-03

**Authors:** Tan Leng Goh, Chee Hoi Leong, Timothy A Brusseau, James Hannon

**Affiliations:** 1 Department of Physical Education and Human Performance, Central Connecticut State University, New Britain, CT 06053, USA; c.leong@ccsu.edu; 2 Department of Kinesiology, University of Utah, Salt Lake City, UT 84112, USA; tim.brusseau@utah.edu; 3 College of Education, Health and Human Services, Kent State University, Kent, OH 44240, USA; jhannon5@kent.edu

**Keywords:** elementary school, body mass index, movement integration, physical activity programs

## Abstract

A classroom-based physical activity curriculum offers an opportunity for students to be active during the school day to combat declining physical activity levels among this population. The effects of classroom-based physical activity curriculum on children of different weight categories is relatively unknown. Therefore, the purpose of this study was to examine the differences in physical activity levels between male and female students, and between students of different weight categories following participation in a classroom-based physical activity curriculum intervention. A total of 210 3rd to 5th grade (age = 9.1 ± 0.1) students from one U.S. elementary school participated in a 4-week intervention. Students’ physical activity levels were measured using pedometers, quantified by step counts pre- and post-intervention. Results from the study indicated that students’ physical activity levels increased after participation in the intervention; male students’ physical activity levels were higher than female students. Additionally, there was an increase in physical activity levels regardless of weight categories, with students of healthy weight exhibiting the most increase following participation in the intervention. In view of the improvement of children’s physical activity levels following their participation in a classroom-based physical activity curriculum, it is recommended that training and resources be provided for teachers to easily implement the curriculum during the school day.

## 1. Introduction

Children are recommended to engage in at least 60 min of physical activity (PA) per day for health benefits. Unfortunately, only 20% of children report sufficient activity to meet the relevant PA guidelines in the U.S. [1]. Physical inactivity is one of the causes of the obesity epidemic in the U.S. and obesity is the leading cause of heart disease, diabetes, certain cancers, and weight-related health problems [2]. Higher values of body mass index (BMI) among children are associated with declines in physical fitness [3]. Furthermore, a majority of the overweight and obese children are classified as the least fit [3]. Children with a higher BMI tend to be physically inactive compared to those with normal BMI [4]. During the school day, boys are more physically active than girls, and PA engagement among girls significantly decreases between the ages of 9–12 [5].

Increasing PA among children through school wide PA promotion has been gaining momentum in the U.S. To that end, schools are encouraged to implement Comprehensive School Physical Activity Programs (CSPAP) which encompasses quality physical education, before, after, and during school activities, as well as activities that engage school staff, family, and the community [6]. School-based PA interventions have been found to have health benefits, especially in improving cardiorespiratory endurance among children [7]. A strategy to increase PA during the school day is through classroom-based PA curriculum, where teachers lead short bouts of activities either during or between classroom instructions [8]. A classroom-based PA curriculum has been found to be effective in increasing students’ school-day PA levels, on-task behavior, cognition, and academic performance [9,10,11,12].

In view of the prevalence of physical inactivity among youths who are overweight/obese, there is no previous study that has examined the effects of a classroom-based PA curriculum on students’ PA, stratified by weight category. Furthermore, understanding that PA among girls declines over time, it is important to further examine effective strategies to engage girls in PA. Therefore, the purposes of this study were to examine the following: (1) Differences in PA levels between male and female elementary school students, and (2) differences in PA between healthy, overweight, and obese elementary school students following participation in a classroom-based PA curriculum. Our hypotheses were as follows: (1) Males will have higher PA levels than females; both males and females will accumulate more PA following participation in the PA curriculum, and (2) students of healthy weight will have higher PA levels than overweight/obese students; all students regardless of weight category will have an increase in PA following participation in the PA curriculum.

## 2. Materials and Methods

### 2.1. Participants and Settings

Based on convenience sampling, the researchers reached out to a number of local schools and a principal of an elementary school, located in a large southwestern city in the United States, agreed to participate in the study. Demographics of the student population were 43% ethnic minority and 65% on free or reduced lunch. Following recruitment from the researchers, 9 classroom teachers volunteered for the study. A total of 210 school students (92 boys and 118 girls) from 9 classes (3rd to 5th grades) participated in the research. The ages of the students were between 8 and 11 years old (age = 9.1 ± 0.1).

**Figure 1:**
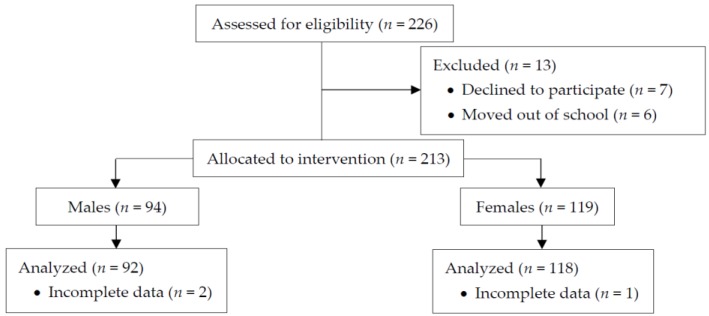
Flow diagram of the progress through the phases of the TAKE 10!^®^classroom-based PA curriculum intervention, presented according to CONSORT statement recommendations [13].

Figure 1 indicates the progress of the participants through the phases of the intervention, according to the Consolidated Standards of Reporting Trials (CONSORT) guidelines [13]. Briefly, students were deemed eligible to participate in the study if they did not possess serious health conditions, injuries, or illnesses that may limit PA participation (<1%). Of the 226 students who were initially recruited, 7 students chose not to participate and 6 students moved out of school. From the remaining 213 students (94 males and 119 females) allocated to the intervention, 3 students (2 males and 1 female) did not have complete data (i.e., missing step count measurements), and hence were excluded in the data analysis. This resulted in a 92.9% participation rate from the students who were initially recruited. Written parental permission and participants’ assent were collected prior to the study. The study was approved by the University of Utah Institutional Review Board (reference number: IRB_00058191).

### 2.2. Measures

The students’ daily in-school PA levels were measured using pedometers (Yamax, CW-600 Digiwalker, Bridgnorth, UK), quantified by the number of step counts recorded during the school day. Pedometers are relatively inexpensive and provide a valid and reliable measurement of students’ PA in school settings [14]. The researchers handed out the pedometers at the start of each school day and collected them at the end of the school day. At the beginning of data collection, the researchers demonstrated the proper wearing of the pedometers and walked around to check that students had securely attached the pedometer to their pants or belts in each classroom. Each pedometer had a number that matched the students’ ID number and students used the same pedometer throughout the study.

The first author and the physical education teacher at the school conducted the measurement of the students’ height and weight in the school’s gymnasium. The students’ body mass index (BMI) was tabulated from their height and weight, using the Centers for Disease Control and Prevention (CDC) formula for calculation [15]. Height was measured (to the nearest 0.1 cm) using a portable free-standing stadiometer. Weight was determined (to the nearest 0.1 kg) using a portable medical scale. BMI status (healthy, overweight, obese) were tabulated from the CDC body mass index-for-age percentiles growth chart [15].

### 2.3. Data Collection and Study Procedures

Prior to the start of the research, the researchers provided a one-hour training for the teachers on the classroom-based PA curriculum, TAKE 10!^®^ (ILSI Research Foundation, Washington, D.C., USA). The TAKE 10!^®^ curriculum consists of 10-minute activities that integrate PA with subjects such as language arts, math, science, social studies, and general health. Teachers were given hands-on practice on implementing the TAKE 10!^®^ activities during the training. During the 4-week intervention, teachers chose an activity from the TAKE 10!^®^ curriculum that complemented the lesson they were teaching each day. The teachers conducted one continuous 10-minute TAKE 10!^®^ activity per day for 4 weeks, either in the morning or afternoon. For example, one teacher conducted an activity with reading where she read a story while students perform the movements in the story (e.g., 17 squats, 11 jumping jacks, etc.). A 4-week intervention was chosen to best accommodate the school’s schedule. In previous studies, a 4-week intervention was a feasible timeline to see improvement in students’ PA levels through the classroom-based PA curriculum [7,12].

Students’ height and weight were collected before the intervention. Students’ pre-PA levels were collected using pedometers the week before the start of the 4-week intervention. Students’ post-PA levels were collected at week 4 of the intervention period. The pedometers were sealed with plastic ties to prevent the students from viewing the number of steps they took, which may alter their activity levels. Students wore the pedometers for 4 consecutive days (Monday to Thursday) during pre- and post-intervention data collection to provide a valid estimate of their daily step counts [16]. Pedometer data were not collected on Fridays because the school had earlier dismissals for the students on Fridays.

### 2.4. Data Analysis

The researchers recorded students’ step counts from the pedometers that were returned each day from the classrooms. The researchers entered the data and generated the results using SPSS (Version 25.0, Chicago, IL, USA). Daily in-school PA levels were quantified as the average number of steps recorded by the pedometers at pre- and post-intervention. Students who recorded step counts above 1000 and below 30,000 for at least one day of pedometer use were kept for analysis [16].

A 2 (sex) × 2 (time) mixed repeated measures analysis of variance (ANOVA) was performed to examine the differences in steps between males and females pre- versus post-intervention. A 3 (BMI status) × 2 (time) mixed repeated measures ANOVA was also performed to examine the differences in steps between children with healthy, overweight, and obese BMI pre- versus post-intervention. If the ANOVAs revealed a significant main effect and/or interaction, post-hoc analyses (Bonferroni) were performed to determine which conditions differed. Effect sizes (*ES*) were generated and *ES* magnitudes of 0.10, 0.30, and 0.50, were interpreted as small, medium, and large effects, respectively [17]. Values are reported as mean ± standard deviation (SD) and the alpha level was set at 0.05.

## 3. Results

Descriptive statistics of anthropometric and physical indictors of the participants are presented in Table 1.

**Figure 2:**
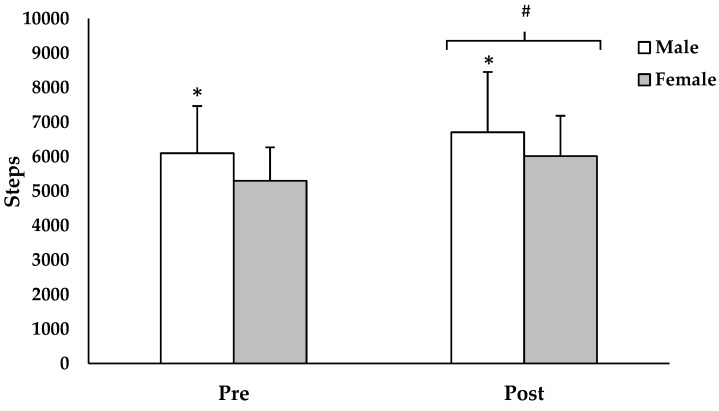
Changes in step count between males and females, following 4 weeks of the TAKE 10!^®^ curriculum intervention. Values are presented as mean ± SD. * Greater compared to female (*p* < 0.001). # Greater compared to pre-intervention (*p* < 0.001).

The repeated measure ANOVA revealed a statistically significant main effect of time in steps for both males and females (*F*(1208) = 59.453, *p* < 0.001; Figure 2). Specifically, the males and females exhibited a 12 ± 19% (6097 ± 1368 pre-intervention vs. 6705 ± 1746 post-intervention; *p* < 0.001, *ES* = 0.44) and 15 ± 22% (5299 ± 971 pre-intervention vs. 6015 ± 1169 post-intervention; *p* < 0.001, *ES* = 0.73) increase in step count following the 4-week TAKE 10!^®^ curriculum intervention, respectively (Figure 2). The repeated measure ANOVA also revealed a statistically significant main effect of sex (*F*(1208) = 21.289, *p* < 0.001), indicating that the males had higher step counts compared to the females at pre-intervention (6098 ± 1368 vs. 5299 ± 971; *p* < 0.001, *ES* = 0.35) and post-intervention (6705 ± 1746 vs. 6015 ± 1169; *p* < 0.005, *ES* = 0.26). There was no significant sex × time interaction (*F*(1208) = 0.394, *p* = 0.531).

**Figure 3:**
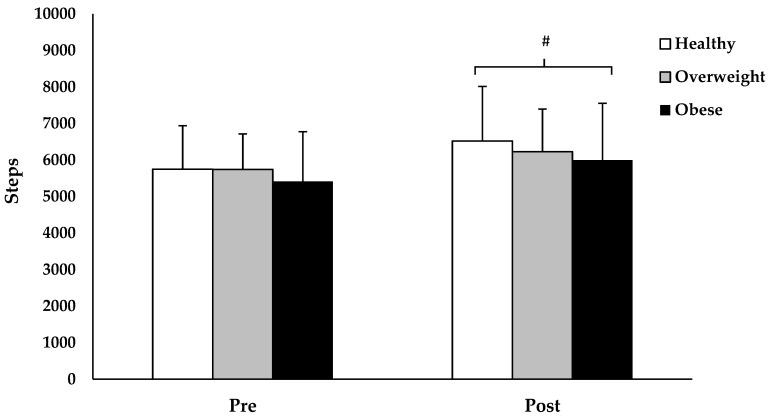
Changes in step count across BMI status, following 4 weeks of the TAKE 10!^®^ curriculum intervention. Values are presented as mean ± SD. # Greater compared to pre-intervention (*p* < 0.001).

The repeated measure ANOVA revealed a statistically significant main effect of time in steps across all BMI statuses (*F*(1207) = 42.082, *p* < 0.001; Figure 3). Specifically, children with healthy BMI exhibited a 16 ± 27% (5748 ± 1190 pre-intervention vs. 6520 ± 1493 post-intervention; *p* < 0.001, *ES* = 0.65), overweight BMI children exhibited a 10 ± 26% (5742 ± 1057 pre-intervention vs. 6226 ± 1265 post-intervention; *p* = 0.012, *ES* = 0.41), and obese BMI children exhibited a 15 ± 26% (5411 ± 1361 pre-intervention vs. 5996 ± 1555 post-intervention; *p* < 0.001, *ES* = 0.43) increase in step count following the 4-week TAKE 10!^®^ curriculum intervention. The main effect of BMI status (*F*(2207) = 2.513, *p* = 0.083) and BMI status × time interaction (*F*(2207) = 0.941, *p* = 0.392) were not statistically significant.

## 4. Discussion

A purpose of this study was to examine differences in PA levels between male and female elementary school students following participation in a classroom-based PA curriculum. Our main finding was that both male and female students increased their PA levels following their 4-week participation in the TAKE 10!^®^ curriculum, with males accumulating significantly more PA during the school day, compared to females. Integrating a technology-based classroom-based PA curriculum, such as GoNoodle, may appeal to female students as a potential strategy to increase their in-school PA levels [18]. As with previous findings [12,19,20,21], the results of this study suggest that a classroom-based PA curriculum is effective in increasing students’ school-day PA levels. The increase in students’ PA levels following their participation in the TAKE 10!^®^ curriculum can likely be attributed to students’ interest in the novelty of the TAKE 10!^®^ curriculum, which took the monotony out of a long classroom instruction period. Indeed, findings from our previous work indicated that students’ enjoyment of their participation in the TAKE 10!^®^ curriculum, translated to improving the buy-in from the teachers to implement a classroom-based PA curriculum within the school day [18]. Most importantly, an increase in PA level can also elicit an improvement to their fitness levels [22]. Hayes and colleagues (2015) reported that as little as 6 min of PA per day could produce a significant improvement in students’ fitness levels over time [22]. Furthermore, long-term participation (more than 3 years of at least 75 min per week) in a classroom-based PA curriculum may prevent the increase in BMI among children and youth [23]. Based on previous research, children’s participation in a classroom-based PA curriculum can potentially influence their PA levels, cardiorespiratory endurance, on-task behavior, cognition, and academic performance positively [7,8,9,10,11,12].

Results from our study also indicated that students’ PA levels increased across all weight categories following participation in the TAKE 10!^®^ curriculum, with no significant differences in PA levels between the different weight categories. Specifically, students with healthy, overweight, and obese BMI exhibited a 16%, 10%, and 15% increase in daily in-school step counts, respectively, following their participation in the TAKE 10!^®^ curriculum. Although students with higher BMI tend to be less physically active, the difference between weight categories in our study were not significant, which is of contrast to a previous study [4]. While Donnelly and colleagues found that children’s participation in classroom-based PA curriculum could prevent an increase in their BMI, it is unknown the effect between children of differing BMI status [23,24]. Therefore, our study adds to the literature by examining if a classroom-based curriculum could improve the PA levels of students of different weight categories. Consequently, the results of this study provide a promising strategy using a classroom-based PA curriculum to promote PA among students, especially among overweight/obese children. Additionally, extending classroom-based PA curriculum to include direct parental involvement could further improve children’s weight status, physical activity, and sedentary behavior [25].

A limitation of the study is that a pre-post design on an intervention group was used in this study. Future studies could include a control group to further examine the effectiveness of the classroom-based PA curriculum on students’ PA levels. Another limitation is the study included participants from one elementary school in the southwestern U.S., and hence results may not be generalizable to and are not representative of other populations of children and youths. A final limitation is that PA levels during the school day were measured in this study, and hence students’ whole day (including out of school PA), as well as long-term changes in PA, could not be ascertained. Future studies may consider exploring the effects of a classroom-based PA curriculum on secondary level students, as well as students from other populations. Furthermore, a longer length of intervention may be implemented to examine if the students will continue to enjoy the novel aspect of the curriculum and whether the teachers will continue implementing the program throughout the school year.

## 5. Conclusions

The findings of this study suggest that male and female children, regardless of their BMI status experienced an increase in their PA levels following their participation in a 4-week classroom-based PA curriculum. In view of children with higher BMI, as well as female children being typically less active than their counterparts, it is important to provide teachers with the resources to competently implement a classroom-based PA curriculum as a potential strategy to increase children’s PA in schools. The TAKE 10!^®^ curriculum used in this study is a useful and effective resource for the teachers. As teachers become more confident in implementing classroom-based PA curriculum, they can adapt and modify the existing resources to fit the curriculum they are teaching each day. It is also important for university teacher-preparation programs to educate preservice teachers on classroom-based PA curriculums and prepare them to competently implement them in schools in the future. Consequently, teachers who have been trained and equipped with resources can easily implement a classroom-based PA curriculum during the school day, thereby increasing PA among children.

## Figures and Tables

**Table 1:** Descriptive statistics of anthropometric and physical fitness indicators. Data is present as mean ± standard deviation (SD).

Measure	Male (*n* = 92)	Female (*n* = 118)	*p*-Value
Age (years)	9.09 ± 0.10	9.02 ± 0.08	0.568
Height (m)	1.40 ± 0.09	1.38 ± 0.09	0.140
Mass (kg)	39 ± 12	36 ± 10	0.034
BMI (kg/m^2^)	19.5 ± 4.2	18.4 ± 3.6	0.044

Note: Independent *t*-test comparing means of males and females.
